# Cognitive fluctuations in Parkinson’s disease dementia: blood pressure lability as an underlying mechanism

**DOI:** 10.1186/s40734-018-0068-4

**Published:** 2018-02-13

**Authors:** David E. Riley, Alberto J. Espay

**Affiliations:** 1 Department of Medical Education, InMotion, Warrensville Heights, OH USA; 2 UC Gardner Neuroscience Institute and Gardner Family Center for Parkinson’s Disease and Movement Disorders, Department of Neurology, University of Cincinnati, 260 Stetson St, Ste 2300, Cincinnati, OH 45267-0525 USA

**Keywords:** Parkinson’s disease dementia, Dementia with Lewy bodies, Cognitive fluctuations, Orthostatic hypotension, Dysautonomia

## Abstract

**Background:**

Cognitive fluctuations refer to alterations in cognition, attention, or arousal occurring over minutes to hours, most commonly in patients with dementias associated with advanced Lewy body pathology. Their pathophysiologic underpinning remains undetermined.

**Case presentation:**

We documented serial blood pressure (BP) measurements in an 86-year-old man with Parkinson’s disease dementia experiencing cognitive fluctuations during an office visit. This patient’s associated dysautonomia included labile BP with orthostatic hypotension and nocturnal hypertension. A spontaneous episode of unresponsiveness occurred while his BP was 72/48. His mental status began to recover immediately as his BP increased to 84/56 when he was placed in a recumbent position; it fully returned to baseline when it reached 124/66 within 1 min. His heart rate remained in the mid-to-high 60s throughout. Subsequent treatment with midodrine markedly reduced the frequency of cognitive fluctuations.

**Conclusions:**

Paroxysmal hypotension may represent an explanatory mechanism for cognitive fluctuations, a common clinical feature in patients with Parkinson’s disease dementia and dementia with Lewy bodies.

## Background

Cognitive fluctuations are a distinctive diagnostic feature of dementia with Lewy bodies (DLB) [1], enshrined as a core feature of clinical diagnostic criteria [2]. These fluctuations consist of paroxysmal deficits of cognitive function or arousal, or both, alternating with alertness and lucidity. “Episodes of going blank or ‘switching off’” have been considered “particularly suggestive of DLB” [3] and of the related phenotype, Parkinson’s disease dementia (PDD). The pathophysiology of cognitive fluctuations remains unknown. We present a patient with PDD in whom cognitive fluctuations were chronologically linked to blood pressure (BP) fluctuations, and suggest that paroxysmal hypotension may represent an explanatory mechanism for episodic confusion in DLB/PDD.

## Case presentation

This 86-year-old man was initially evaluated 4 years earlier for a 5-month history of a soft voice, drooling, a right thumb tremor, and difficulty arising from chairs and walking. Examination revealed additional findings of activated rigidity and mild akinesia in the right upper limb, and micrographia. Gait was slightly slow. Postural stability was normal. Notably, BP was 194/107 mmHg supine with a pulse of 81; 30 min later sitting BP and pulse were 187/121 mmHg and 76. He was diagnosed with Parkinson’s disease (PD) and responded to carbidopa/levodopa 25/100, up to 2 tablets t.i.d. Tremor, mobility, gait and drooling all improved; his voice did not. Two years after symptom onset, he had several syncopal episodes. Tilt table testing documented an asymptomatic progressive drop in BP from 168/93 mmHg supine to 75/55 mmHg upright by minute 38. At 3 years, he and his wife reported difficulty with “finding the right words” while speaking, dialing a telephone, forgetfulness, confusion regarding dates, and visual hallucinations of people or animals. His Montreal Cognitive Assessment score was 22/30; he lost 3 points for delayed recall, 2 points for his drawing of a clock (neither the numbers nor the hands were correct), and 1 point each for failing the trails test, inability to copy a cube, and impaired semantic fluency, and recalled only 2 of 5 objects. He later had to give up driving and leadership roles in community organizations. He wore adult diapers for urinary incontinence. At the time the events below occurred and relative to the cognitive features, motor function remained relatively intact, apart from moderate hypophonia.

His wife reported observing increasingly frequent episodes of unresponsiveness. She described these episodes beginning with a tired appearance and yawning, followed by a “glazed” or “glassy” look on his face and immobile unresponsiveness that could last up to 10 min. These had first been seen while standing 18 months after his initial disease-related symptoms, but now occurred predominantly while seated, often after eating breakfast. Following a recent episode, he had appeared “clammy” and was admitted to hospital, where his systolic BP soared as high as 250 mmHg. He was discharged on 3 antihypertensive medications (lisinopril, hydralazine, metoprolol).

His wife had learned that getting him to lie down would allow him to recover promptly from an episode of unresponsiveness. Whenever he entered an unresponsive state, she summoned attendants in their residence, who placed him horizontally on the floor and elevated his legs. Within seconds, he would become responsive again.

As she related this history during the office visit, she commented “he is going into one”, which she could “just tell” by his facial expression. He remained seated with his eyes open, but was unresponsive to questions and did not react to simple commands. His BP was 72/48 mmHg with a pulse of 70. He was then placed supine on the floor, head on a pillow and feet on a chair. He immediately became conversant and able to follow commands. His BP was 84/56 mmHg. He stated he felt “fine”. After 1 min, his BP rose to 124/66 mmHg with a pulse of 69. After 3 min in the same position his BP was 138/71 mmHg with a pulse of 61. He was subsequently able to return to a seated position while maintaining his alert and conversant status.

His BP medications were gradually withdrawn. He was ultimately stabilized on a combination of midodrine 2.5 mg t.i.d. (last dose at 16:00) and hydralazine 50 mg at bedtime; without the latter, his nocturnal systolic BP was routinely greater than 200 mmHg. On this regimen, the frequency of his unresponsive episodes markedly decreased from nearly daily to being spaced several months apart. However, close monitoring revealed he continued to experience labile BP. Two years after the incident recorded above, a nurse reported a BP of 70/41 mmHg during an unresponsive episode. On one occasion, his seated BP had been 200/110 mmHg at 08:00, then 96/68 mmHg at 10:00. Shortly afterward, a nurse accompanying him during a week of travel noted widely varying BPs ranging from a low of 96/68 mmHg to a high of 208/124 mmHg.

## Discussion

This patient had clinical PDD combining early classic motor PD, followed by dementia with hallucinations, and dysautonomia, manifested most prominently by labile BP expressed as sitting and orthostatic hypotension (OH), sitting and supine hypertension (SH), and nocturnal hypertension (NH). He exhibited typical cognitive fluctuations consisting of unresponsive episodes, which were reversible upon assumption of a supine position. In one documented episode, the patient’s BP was low, and his mental status recovered when his BP was raised by a postural change. Episodes largely resolved when he was treated with midodrine. The clinical evidence in this case suggests that bouts of hypotension, some of them possibly postprandial or drug-induced, were responsible for the patient’s cognitive fluctuations.

Ambulatory blood pressure monitoring (ABPM) has opened a window into the unstable cardiovascular environment of PD [4–7] that provides a plausible mechanism for patient’s cognitive fluctuations. Clinical expressions of disordered BP homeostasis noted in ABPM studies include OH [5, 7], postprandial hypotension [5, 6], NH [5–7] and BP lability [5–7]. Variations in BP in patients with PD, PDD, and DLB are common and can be profound. One ABPM study documented BP fluctuations of over 100 mmHg within a 24 h period in 67.6% of patients with PD [6]. In retrospect, in one of the earliest studies of dysautonomia in PD, Rajput and Rozdilsky may have described a case similar to ours. One of their three patients subjected to intraarterial BP recordings had a history of “intermittent confusion” and showed unexplained BP fluctuations between 90/58 and 230/130 mmHg [8].

Chronic cognitive impairment has been linked to hypotension in PD [9, 10]. While Allcock and colleagues postulated that the appearance of OH in PD is a marker for disease progression with spread of Lewy body pathology [9], Udow and colleagues suggested that OH may also directly exacerbate the symptoms arising from a diseased brain through repeated episodes of cerebral hypoperfusion [10].

A few investigators have studied the acute effects of hypotension on cognitive performance. Peralta and colleagues found that attention, though not word fluency, decreased in tandem with BP on tilt-table testing in patients with PDD [11]. Poda and colleagues examined the effects of posture on cognitive function in 10 patients with OH (only one with PD) [12]; orthostatic challenge produced deficits in global cognitive and executive function. Centi and colleagues determined, in a tilt-table study, that non-demented patients with PD demonstrated cognitive declines when examined in the upright position compared to lying supine [13]. Deterioration was greater in those with OH than those without. Thus, acute declines in BP have been linked to transient declines in cognitive performance in patients with PD, with or without dementia.

**Fig. 1:**
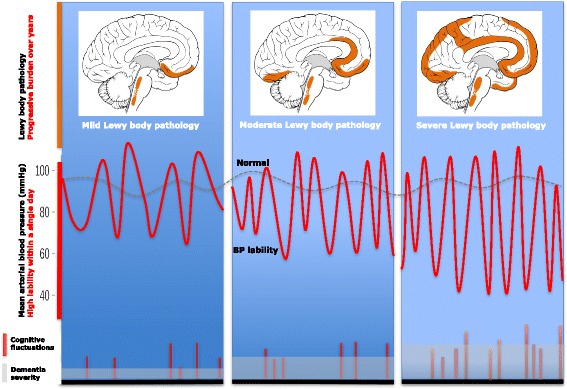
Theoretical relationship between blood pressure lability and cognitive fluctuations according to burden of Lewy body pathology. The undulating red line represents the erratic ambulatory blood pressure recording of a patient with a Lewy body disorder and dysautonomia over the waking hours of a single day, with BP wandering between and over thresholds of hypotension and hypertension. The blue panels represent mild, moderate, and severe Lewy body pathology. As the BP lability increases with disease progression so does the frequency of hypotension-induced cerebral hypoperfusion episodes, which may correlate with cognitive fluctuations. Although this has not been directly examined, we propose that with severe cortical pathology (right-most panel), the cognitive fluctuations may become subclinical, obscured by a higher baseline dementia, which precludes inter-episode lucidity

In this context, the cognitive fluctuations in the present case can be seen as a partial form of syncope without muscle atonia. Although our observations are based on a single case, we speculate that paroxysmal hypotension may underlie cognitive fluctuations in other patients. The BP lability in ABPM studies noted above [5–7] can produce, within a single day, bouts of hypertension or hypotension, the latter representing a viable mechanism for these episodes. Further, clinician attempts to avoid episodes of supine hypertension may increase the prevalence and severity of OH-related disability. What may have previously prevented the recognition of this relationship has been the intuitive nosological classification of cognitive fluctuations in the cognitive domain of nonmotor manifestations of PD, rather than as a manifestation of dysautonomia-related BP dysregulation. Dynamic changes in BP can account for the abrupt and transient nature of cognitive fluctuations more readily than the distribution or magnitude of cortical pathology. However, the strong association of such fluctuations with dementia suggests that crossing the threshold into unresponsiveness or confusion requires underlying cognitive compromise (Fig. 1). Since cognitive fluctuations are recognized by the periods of lucidity between them, it is conceivable that, despite ongoing BP lability, fluctuations may become less apparent, obscured by a progressively greater baseline dementia that lowers the ceiling of lucidity.

## Conclusions

We suggest that cognitive fluctuations in PDD and DLB may be associated with cardiovascular instability, which is a common comorbidity in PDD and DLB [14, 15] compared to Alzheimer's disease and other types of dementia [1]. The effect of hypotension may be similar (or complementary) to other destabilizing variables on brain function when buffering capacity is limited, such as infections, electrolyte imbalance, or iatrogenic effects. ABPM studies will be needed to further examine the relationship between paroxysmal hypotension and cognitive fluctuations. ABPM studies should also explore the safe range of supine hypertension necessary to minimize bouts of hypotension, if confirmed as a major trigger for paroxysmal cognitive fluctuations, and as a contributor to chronic cognitive decline.

## Abbreviations

ABPM: Ambulatory blood pressure monitoring
DLB: Dementia with Lewy bodies
NH: Nocturnal hypertension
OH: Orthostatic hypotension
PD: Parkinson’s disease
PDD: Parkinson’s disease dementia
SH: Supine hypertension

## References

[1] Ferman TJ, Smith GE, Boeve BF (2004). DLB fluctuations: specific features that reliably differentiate DLB from AD and normal aging. Neurology.

[2] McKeith IG, Boeve BF, Dickson DW (2017). Diagnosis and management of dementia with Lewy bodies: fourth consensus report of the DLB consortium. Neurology.

[3] McKeith IG, Galasko D, Kosaka K (1996). Consensus guidelines for the clinical and pathologic diagnosis of dementia with Lewy bodies (DLB): report of the consortium on DLB international workshop. Neurology.

[4] Brevetti G, Bonaduce D, Breglio R (1990). Parkinson's disease and hypotension: 24-hour blood pressure recording in ambulant patients. Clin Cardiol.

[5] Senard JM, Chamontin B, Rascol A, Montastruc JL (1992). Ambulatory blood pressure in patients with Parkinson's disease without and with orthostatic hypotension. Clin Auton Res.

[6] Tsukamoto T, Kitano Y, Kuno S (2013). Blood pressure fluctuation and hypertension in patients with Parkinson's disease. Brain Behav.

[7] Vichayanrat E, Low DA, Iodice V, Stuebner E, Hagen EM, Mathias CJ (2017). Twenty-four-hour ambulatory blood pressure and heart rate profiles in diagnosing orthostatic hypotension in Parkinson's disease and multiple system atrophy. Eur J Neurol.

[8] Rajput AH, Rozdilsky B (1976). Dysautonomia in parkinsonism: a clinicopathological study. J Neurol Neurosurg Psychiatry.

[9] Allcock LM, Kenny RA, Mosimann UP (2006). Orthostatic hypotension in Parkinson's disease: association with cognitive decline?. Int J Geriatr Psychiatry.

[10] Udow SJ, Robertson AD, MacIntosh BJ (2016). ‘Under pressure’: is there a link between orthostatic hypotension and cognitive impairment in α-synucleinopathies?. J Neurol Neurosurg Psychiatry.

[11] Peralta C, Stampfer-Kountchev M, Karner E (2007). Orthostatic hypotension and attention in Parkinson’s disease with and without dementia. J Neural Transm.

[12] Poda R, Guaraldi P, Solieri L (2012). Standing worsens cognitive functions in patients with neurogenic orthostatic hypotension. Neurol Sci.

[13] Centi J, Freeman R, Gibbons CH, Neargarder S, Canova AO, Cronin-Golomb A (2017). Effects of orthostatic hypotension on cognition in Parkinson disease. Neurology.

[14] Thaisetthawatkul P, Boeve BF, Benarroch EE (2004). Autonomic dysfunction in dementia with Lewy bodies. Neurology.

[15] Poewe W (2007). Dysautonomia and cognitive dysfunction in Parkinson's disease. Mov Disord.

